# Serotype distribution of invasive, non-invasive and carried *Streptococcus pneumoniae* in Malaysia: a meta-analysis

**DOI:** 10.1186/s41479-021-00086-7

**Published:** 2021-05-25

**Authors:** Alex J. J. Lister, Cheng Foh Le, Eddy Seong Guan Cheah, Mohd Nasir Mohd Desa, David W. Cleary, Stuart C. Clarke

**Affiliations:** 1 Faculty of Medicine and Institute for Life Sciences, Infectious Disease Epidemiology Group, University of Southampton, Mailpoint 814, Level C, Sir Henry Wellcome Laboratories, South Block, University Hospital Southampton Foundation NHS Trust, Southampton, SO16 6YD UK; 2 School of Biosciences, Faculty of Science and Engineering, University of Nottingham Malaysia, Jalan Broga, 43500 Semenyih, Selangor Malaysia; 3 Department of Biological Science, Faculty of Science, Universiti Tunku Abdul Rahman (UTAR), Kampar Campus, 31900 Kampar, Perak Malaysia; 4 Department of Biomedical Sciences, Faculty of Medicine and Health Sciences, Universiti Putra Malaysia, 43400 Serdang, Selangor Malaysia; 5 NIHR Southampton Biomedical Research Centre, University Hospital Southampton Foundation NHS Trust, Southampton, UK; 6 Global Health Research Institute, University of Southampton, Southampton, UK; 7 Institute for Research, Development and Innovation, International Medical University, Kuala Lumpur, Malaysia

**Keywords:** Pneumococcal disease, National Immunisation Programme, Vaccine types, PCV10, PCV13, Pneumonia, Vaccine, Serotype, Antimicrobial resistance

## Abstract

**Background:**

Pneumococcal pneumonia is the leading cause of under-five mortality globally. The surveillance of pneumococcal serotypes is therefore vital for informing pneumococcal vaccination policy and programmes. Pneumococcal conjugate vaccines (PCVs) have been available as an option in the private healthcare setting and beginning December 2020, PCV10 was incorporated as part of routine national immunisation programme (NIP) in Malaysia. We searched existing literature on pneumococcal serotype distribution across Malaysia to provide an overall view of this distribution before the implementation of PCV10.

**Methods:**

Online databases (PubMed, Ovid MEDLINE and Scopus), reference lists of articles identified, and grey literature (Malaysian Ministry of Health website, WHO website) were systematically searched for relevant literature on pneumococcal serotype distribution across Malaysia up to 10th November 2020. No lower date limit was set to maximise the number of target reports returned. Results of serotypes were split by age categories, including ≤5 years, > 5 years and unreported for those that did not specify.

**Results:**

The search returned 18 relevant results, with a total of 2040 isolates. The most common serotypes across all disease types were 19F (*n* = 313, 15.3% [95%CI: 13.8–17.0]), 23F (*n* = 166, 8.1% [95%CI: 7.0–9.4]), 14 (n = 166, 8.1% [95%CI: 7.0–9.4]), 6B (*n* = 163, 8.0% [95%CI: 6.9–9.2]) and 19A (*n* = 138, 6.8% [95%CI: 5.8–7.9]).

**Conclusion:**

Four of the most common serotypes across all isolate sources in Malaysia are covered by PCV10, while PCV13 provides greater serotype coverage in comparison to PCV10. There is still a need for surveillance studies, particularly those investigating serotypes in children under 5 years of age, to monitor vaccine effectiveness and pneumococcal population dynamic following implementation of PCV10 into routine immunisation.

## Introduction

*Streptococcus pneumoniae* infection remains the leading cause of bacterial pneumonia worldwide but can also lead to other life-threatening invasive pneumococcal diseases (IPDs) such as meningitis and sepsis, as well as non-invasive diseases (non-IPDs) such as sinusitis and otitis media [1]. The causative bacterium typically resides asymptomatically in the upper respiratory tract in the carriage state but can also evade the immune system of the host and cross the mucosal membrane to cause invasive disease [2]. Bacterial transmission occurs through direct contact of respiratory droplets expelled by infected individuals or asymptomatic carriers [3]. The pathogen has a three times higher colonisation prevalence in individuals in low and middle-income countries (LMICs) compared to those in high-income countries [4, 5].

A total of 100 serotypes of *S. pneumoniae* have been identified to date, based on the composition of their polysaccharide capsule [6], which is one of their major virulent factors required for host immune evasion and colonisation of the upper respiratory tract [7].

There are currently two licensed PCV vaccines, Synflorix® (PCV10, GlaxoSmithKline) and Prevnar 13® (PCV13, Pfizer), which contain purified capsular polysaccharides from 10 and 13 pneumococcal serotypes, respectively. PCV10 includes purified capsular polysaccharides from serotypes 1, 4, 5, 6B, 7F, 9 V, 14, 18C, 19F and 23F, whilst PCV13 contains the same but with the addition of purified capsular polysaccharide from serotypes 3, 6A and 19A [8, 9]. Several new pneumococcal vaccine candidates are currently in clinical trials. One of these, Pneumosil® (PCV10, Serum Institute of India Pvt. Ltd.), is in pre-qualification with the World Health Organization [10] whilst PCV15 (Merck Sharp & Dohme Corp., Kenilworth, NJ, USA), which is currently in the Phase III clinical trial, contains the same purified capsular polysaccharides as PCV13 serotypes along with serotypes 22F and 33F. The 20vPnC (PCV20, Pfizer), which is also in Phase III clinical trials, containing the same purified capsular polysaccharides as PCV13 serotypes along with serotypes 8, 10A, 11A, 12F, 15B, 22F and 33F. Both PCV15 and PCV20 have shown consistent safety profiles compared to currently available PCVs [11, 12]. Despite the availability of pneumococcal vaccines, LMICs face barriers to their introduction due to lack of country-specific data on disease burden and circulating serotypes and the cost of the vaccines [13–15].

GAVI, the Vaccine Alliance, provides free access to vaccines for low-income countries but, as an upper-middle-income country, Malaysia is not eligible for this initiative [16, 17]. There has been much delay in implementing pneumococcal vaccination under the Malaysian NIP, lagging behind even the neighbouring Southeast Asian countries, including Laos, Cambodia and the Philippines (some who have received support from GAVI). Although many LMIC countries have introduced PCV, Malaysia had only previously offered PCV as an optional vaccine in the private healthcare sector [18, 19]. Due to the high cost associated with PCVs (about RM1,000 or USD240 for a complete four-dose course of PCV13, which is equivalent to about RM300–350 per dose), only families afford to do so would consider pneumococcal vaccination, therefore resulting in low coverage across the country [14].

There is relatively little information on pneumococcal epidemiology across Southeast Asia and Malaysia, which is concerning due to the rise in antimicrobial resistance and risk associated with pneumococcal infection [16]. In Malaysia, pneumococcal meningitis alone contributes to 2809 cases annually which is significantly higher than those recorded in neighbouring countries Singapore and Thailand [20]. Here we provide an update on the distribution of pneumococcal serotypes from IPD, non-IPD and carriage cases in Malaysia.

## Methods

The online databases PubMed and Scopus were searched for pre-existing literature on pneumococcal serotype prevalence in Malaysia. Reference lists of relevant articles were also searched for relevant articles not found in the database search. No lower date limit was set to maximise the number of potential articles and the search included those published up to 10th November 2020. Search terms included: ‘SE Asia*’, ‘South East Asia*’, Southeast Asia*’, ‘Southeastern Asia*’, ‘*Streptococcus pneumoniae*’, ‘S pneumoniae’, ‘pneumococc*’, ‘pneumonia’, ‘serotype*’, ‘serogroup*’, ‘seroprevalence’ and ‘Malaysia’ separated by the binary operators ‘OR’ and ‘AND’. Article titles and abstracts were searched with these terms to identify potential sources of data. Grey literature (Malaysian Ministry of Health, WHO website) was also searched.

Inclusion criteria included full texts that reported *S. pneumoniae* serotypes in Malaysia, those who reported invasive disease (IPD), non-invasive disease (non-IPD) and carriage studies, as well as those reporting all age groups and genders. Both serogroup and serotype data were included. Exclusion criteria included those studies that were not written in English, non-Malaysian studies, animal studies, case studies, reviews, articles on biochemical techniques or genetics for serotyping, and studies that only reported case numbers or antibody levels without specifying the pneumococcal serotypes.

From the initial search, returned articles were scanned for duplicates, which were then removed. Titles and abstracts were scrutinised, and any articles deemed irrelevant were removed. The full texts of the remaining articles were then reviewed based on the inclusion/exclusion criteria. Of the eligible articles, data were extracted and summarised in a single table. Serotypes were also grouped into age categories, including under 5 years, over 5 years and unreported. The unreported category represents the ages that were not disclosed in the studies or did not fit the age groupings.

Serotype data were grouped into three categories, including IPD, non-IPD and carriage. Within these categories, serotypes were categorised further into vaccine types (VTs), which were those serotypes found in the PCVs, vaccine-related types (VRT), non-vaccine types (NVTs), non-typeable (NT) strains, which were ‘unencapsulated’ serotypes, and unknown (N/A) serotypes, which are those that could not be determined through conventional methods or from studies that did not explicitly report the specific serotypes. Serotype prevalence with 95% confidence intervals was calculated using Microsoft Excel 2019. Graphical representation of serotype distribution was performed using Prism 8 for Windows (GraphPad Software, Inc.).

## Results

**Fig. 1:**
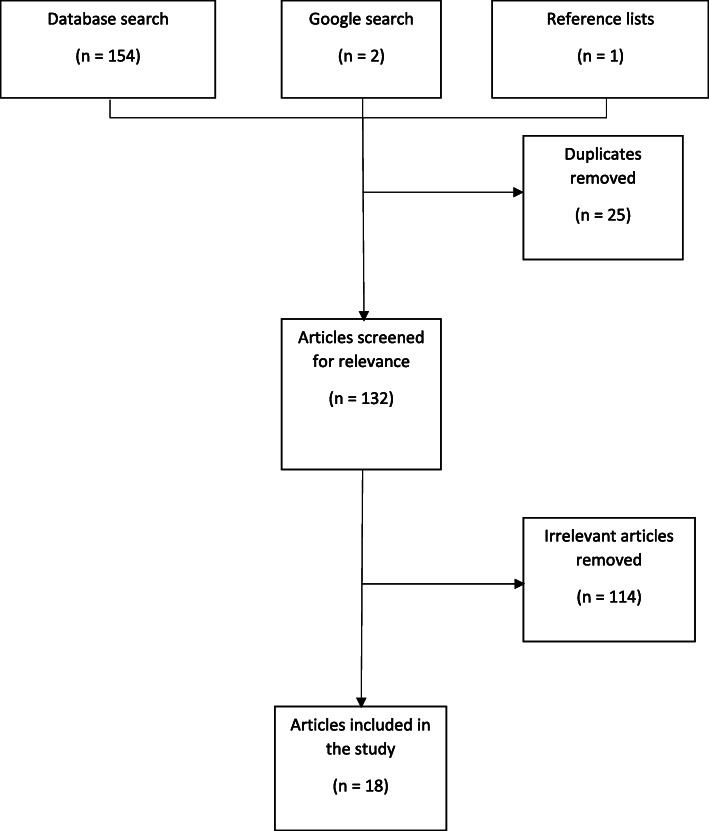
PRISMA flowchart of identified articles reporting pneumococcal serotypes in Malaysia

One hundred and fifty-four articles were returned from the database search (Fig. 1), 25 of which were duplicates, with one article identified from the reference lists and two from the Google search. No articles were identified from the grey literature. The final analysis included 18 articles (Table 1), with data collected from 1989 to 2017. Eleven of the 18 studies analysed included isolates from invasive disease. Serotype data from a total of 2040 pneumococcal isolates were extracted from the articles and separated by source category and by the age of the cases, with 1008 isolates from IPD, 583 from non-IPD and 299 from carriage (Table 2).

**Table 1:** Articles identified from review containing relevant pneumococcal serotype data in Malaysia

No.	Article title (year)	Collection period	No. of isolates	IPD / non-IPD / Carriage	Age range	Source	Serotyping method	Location	Serotypes identified
1	Antibiotic susceptibility and serotype distribution of *Streptococcus pneumoniae* in Malaysian children [21]	Sep 1984 -Aug 1985	92	Non-IPD	1 month - < 5 years	Nasopharyngeal aspirates	Quellung	Three unknown hospitals in Kuala Lumpur, Ipoh and rural Kota Bahru	6A, 6B, 10A, 11A, 13, 14, 15B, 15C, 16, 18A, 18C, 19A, 19B, 19F, 20, 23A, 23F, 34, 39, ‘rough’
2	Genotypic characterisation of Malaysian human isolates of *Streptococcus pneumoniae* from carriage and clinical sources [22]	1989–2012 Carriage in 2010	107	Carriage and IPD	< 5 years - > 50 years	Anterior nares, blood, CSF, pleural fluid, sputum, ear, eye, pus, throat, nasopharyngeal fluid, others (unknown)	Multiplex PCR	Unknown hospital locations and communities	1, 4, 6A, 6B, 7A/F, 11A/D, 12F, 14, 15B/C, 17, 18A/B/C, 19A, 19F, 23A, 23F, NT
3	Epidemiology of *Streptococcus pneumoniae* infection in Malaysia [23]	Oct 1995 - Sep1996	201	IPD and non-IPD	< 5 years, > 5 years	Sputum, blood, CSF, body fluid, pus	Pneumotest	Hospital Pulau Pinang, Hospital Kuala Lumpur, Hospital Sultanah Aminah (Johor Bahru, Johor), Hospital Queen Elizabeth (Kota Kinabalu, Sabah), University of Malaya Medical Centre (Kuala Lumpur)	1, 2, 3, 4, 5, 6A, 6B, 6C, 7A, 7B, 7C, 7F, 8, 9A, 9 L, 9 N, 10A, 11A, 11C, 11F, 12A, 12F, 14, 15B, 17A, 18A, 18B, 18C, 18F, 19A, 19B, 19C, 19F, 20, 22A, 22F, 23, 23A, 23B, 23F, NA
4	Spread of drug-resistant *Streptococcus pneumoniae* in Asian countries: Asian Network for Surveillance of Resistant Pathogens (ANSORP) Study [24]	Sep 1996 - Jun 1997	2	IPD	Unknown	Blood, CSF, ascites, pleural fluid, synovial fluid, sinus aspirate	Quellung	Universiti Kebangsaan Malaysia, Bangi, Selangor	19, 23
5	Carriage of Antibiotic-Resistant Pneumococci among Asian Children: A Multinational Surveillance by the Asian Network for Surveillance of Resistant Pathogens (ANSORP) [25]	Jan 1998 -Mar 1999	58	Carriage	< 5 years	Asymptomatic carriage	Quellung	Universiti Kebangsaan Malaysia, Kuala Lumpur	1, 3, 4, 5, 6, 7, 9, 14, 15, 19, 23, Other (unknown)
6	Penicillin susceptibility and molecular characteristics of clinical isolates of *Streptococcus pneumoniae* at the University of Malaya Medical Center, Kuala Lumpur, Malaysia [26]	Mar1999 -Jul 2000	50	IPD and non-IPD	< 13 years, > 13 years	Sputum, NPS, tracheal, throat, blood, CSF, pleural fluid, eye, ear, vagina.	Pneumotest	University of Malaya Medical Centre, Kuala Lumpur	1, 5, 6A, 7B/C, 14, 15A, 15C, 19F, 23A, 23B, 23F, 16/36/37, NT
7	Capsular Serotype and Antibiotic Resistance of *Streptococcus pneumoniae* Isolates in Malaysia [27]	Mar1999 -Feb 2007 (except 2001, 2004)	151	IPD and non-IPD	< 5 years, 5–59 years, > 60 years	Blood, CSF, nasopharyngeal secretion, tracheal secretion, sputum, bronchoalveolar lavage, others	Quellung	University of Malaya Medical Centre, Kuala Lumpur	1, 3, 6A/6B, 7C/7B/40, 7F/7A, 9 V/9A, 10A, 11A/11D, 12F/12A, 14, 15A, 15B/15C, 16F, 18C/18B/18A/18F, 19A, 19F, 23F, 35B, 35F/47F, 34
8	High prevalence of antimicrobial resistance among clinical *Streptococcus pneumoniae* isolates in Asia (an ANSORP Study) [28]	Jan 2000 -Jun 2001	41	IPD	< 5 years and adults	Blood, CSF, ascitic fluid, pleural fluid, synovial fluid, sinus aspirate, middle ear aspirate	Quellung	Universiti Putra Malaysia, Serdang, Selangor and Universiti Malaya, Kuala Lumpur	4, 6A, 6B, 14, 19A, 19F, 23A, 23F, Others (unknown)
9	Novel clones of *Streptococcus pneumoniae* causing invasive disease in Malaysia [29]	Feb 2000 – Jan 2007	30	IPD	< 15 years, > 15 years, unknown age	Blood, CSF, pleural fluid, pericardial fluid	Multiplex PCR	University of Malaya Medical Centre, Kuala Lumpur	3, 4, 6A, 6B, 9 N, 9 V, 11A, 14, 15A, 16F, 18C, 19A, 19F, 23F, 34
10	Current trend of pneumococcal serotypes distribution and antibiotic susceptibility pattern in Malaysian hospitals [30]	Jan 2008 - Dec 2009	443	IPD and non-IPD	< 1 year - > 60 years	Blood, CSF, other sterile sites, sputum, eyes, ear, vagina, wounds	Pneumotest	Hospital Ampang, Ampang Jaya, Selangor Hospital Selayang, Batu Caves, Selangor Hospital Sungai Buloh, Sungai Buloh, Selangor Hospital Tengku Ampuan Rahimah, Klang, Selangor Hospital Pulau Pinang Hospital Sultanah Bahiyah, Alor Setar, Kedah Hospital Raja Permaisuri Bainun, Ipoh, Perak Hospital Sultanah Aminah, Johor Bahru, Johor Hospital Tuanku Ja’afar, Seremban, Negeri Sembilan Hospital Tengku Ampuan Afzan, Kuantan, Pahang Hospital Sultanah Nur Zahira, Kuala Terengganu, Terengganu Hospital Raja Perempuan Zainab II, Kota Bharu, Kelantan Hospital Umum Sarawak, Kuching, Sarawak Hospital Queen Elizabeth, Kota Kinabalu, Sabah Hospital Kuala Lumpur University of Malaya Medical Centre, Kuala Lumpur Institute for Medical Research, Kuala Lumpur	1, 3, 4, 5, 6A, 6B, 7F, 14, 18C, 19A, 19F and 23F
11	Changing trends in antimicrobial resistance and serotypes of *Streptococcus pneumoniae* isolates in Asian countries: an Asian Network for Surveillance of Resistant Pathogens (ANSORP) Study [31]	Mar 2008 - Dec 2009	165	IPD	Unknown	Throat swab, nasal swab, nasopharyngeal aspirate	Quellung	Hospital Selayang, Batu Caves, Selangor Hospital Sungai Buloh, Sungai Buloh, Selangor Hospital Pulau Pinang Hospital Sultanah Aminah, Johor Bahru, Johor Hospital Tuanku Ja’afar, Seremban, Negeri Sembilan Hospital Sultanah Nur Zahira, Kuala Terengganu, Terengganu Hospital Raja Perempuan Zainab II, Kota Bharu, Kelantan Hospital Queen Elizabeth, Kota Kinabalu, Sabah Hospital Kuala Lumpur University of Malaya Medical Centre, Kuala Lumpur Institute for Medical Research, Kuala Lumpur	1, 3, 5, 6A, 6B, 6C, 7C, 9 V, 10, 11, 14, 15, 17, 18C, 19A, 19F, 23F, 23A, 20, 35, 13/28, 16/36/37, Other (unknown), NT
12	Determination of phenotypes and pneumococcal surface protein A family types of *Streptococcus pneumoniae* from Malaysian healthy children [32]	Jun 2010 - Dec 2010	69	Carriage	≤5 years	Asymptomatic carriage	Pneumotest	Three unknown day care centres, Kuala Lumpur	6A, 6B, 11A, 15C, 18C, 19A, 19F, 20, 23A, 23B, 23F, NT
13	Changes in serotype distribution and antimicrobial resistance of *Streptococcus pneumoniae* isolates from adult patients in Asia: Emergence of drug-resistant non-vaccine serotypes [33]	Dec 2012 - Jul 2017	123	IPD	> 50 years	Blood, CSF, pleural fluid	Quellung	39 hospitals in 16 unknown cities	4, 6, 6A, 6B, 6C, 9 V, 10, 11A, 12, 14, 15, 15A, 18C, 19A, 19F, 20, 22F, 23A, 23F, 35B, Others (incl. 6D, 7B, 7C, 11C, 11D, 13/28, 15C, 16/36/37, 18A, 18B, 18F, 19B, 19C, 21, 22A, 23B, 24/31/40, 27/32/41, 38, and 39), NT
14	Serotypes & penicillin susceptibility of *Streptococcus pneumoniae* isolated from children admitted to a tertiary teaching hospital in Malaysia [34]	Jan 2013 - May 2015	125	Carriage	1 month – 12 years	Nasopharyngeal specimens, sputum and bronchoalveolar lavage	Multiplex PCR	University of Malaya Medical Centre, Kuala Lumpur	3, 6A/B, 6C, 11/A/D/F, 15A/F, 19A, 19F, 23A, 23F, 34
15	Molecular detection and genotypic characterisation of *Streptococcus pneumoniae* isolated from children in Malaysia [35]	2013–2015	95	Non-IPD	< 12 years	Nasopharyngeal swab and secretions	Multiplex PCR	University of Malaya Medical Centre, Kuala Lumpur	3, 6A/B, 6C, 9 N/9 L, 11A/D/F, 15A/F, 17A, 19A, 19F, 23A, 23F, 23B, 34, NT
16	Pneumococcal serotype distribution and antibiotic susceptibility in Malaysia: A four-year study (2014–2017) on invasive paediatric isolates [19]	May 2014 - Dec 2017	245	IPD	13 days – 4 years, 364 days	Blood and CSF	Quellung	Multiple hospital locations around Malaysia	1, 3, 4, 6A, 6B, 6C, 8, 11A, 11C, 14, 15A, 15B, 15C, 18C, 19B, 19F, 23A, 23F, NT
17	A study of the prevalence of *Streptococcus pneumoniae* serotypes in patients hospitalised for community-acquired pneumonia [36]	Dec 2014 - Aug 2015	9	Non-IPD	59–76 years	Sputum cultures	PCR	Universiti Kebangsaan Malaysia Medical Center, Kuala Lumpur	8F, 33F
18	The upper respiratory tract microbiome of indigenous Orang Asli in north-eastern Peninsular Malaysia [37]	August 2017	34	Carriage	< 5 years, 5–17 years, 18–65 years	Asymptomatic carriage	Slide agglutination	Kampung Sungai Pergam and Kampung Berua, north-eastern Terengganu	3, 4, 6A, 6B, 6C, 11A, 14, 15B, 18A, 19F, 23A, 23F, 34, 35F, NT

**Table 2:** Serotype counts across carriage, non-IPD and IPD studies

Country	Serotype	Carriage			Non-IPD			IPD			Total Number of isolates per serotype N (%)
		≤5 N (%)	> 5 N (%)	Unreported N (%)	≤5 N (%)	> 5 N (%)	Unreported N (%)	≤5 N (%)	> 5 N (%)	Unreported N (%)	
	**PCV10**										
Malaysia	1	0 (0.0)	0 (0.0)	0 (0.0)	0 (0.0)	3 (1.7)	22 (6.3)	6 (1.8)	14 (4.4)	33 (9.4)	78 (3.8)
	4	0 (0.0)	1 (3.3)	0 (0.0)	0 (0.0)	4 (2.2)	1 (0.3)	2 (0.6)	9 (2.8)	5 (1.42)	22 (1.1)
	5	0 (0.0)	0 (0.0)	0 (0.0)	0 (0.0)	4 (2.2)	0 (0.0)	1 (0.3)	3 (0.9)	7 (2.0)	15 (0.7)
	6B	8 (6.1)	4 (13.3)	5 (2.6)	12 (8.1)	10 (5.6)	16 (4.6)	60 (17.8)	24 (7.5)	24 (6.8)	163 (8.0)
	7F	0 (0.0)	0 (0.0)	0 (0.0)	1 (0.7)	0 (0.0)	1 (0.3)	2 (0.6)	7 (2.2)	3 (0.9)	14 (0.7)
	9 V	0 (0.0)	0 (0.0)	0 (0.0)	0 (0.0)	0 (0.0)	0 (0.0)	1 (0.3)	3 (0.9)	2 (0.6)	6 (0.3)
	14	1 (0.8)	2 (6.7)	1 (0.5)	6 (4.0)	6 (3.4)	8 (2.3)	74 (21.9)	30 (9.4)	38 (10.8)	166 (8.1)
	18C	2 (1.5)	0 (0.0)	0 (0.0)	2 (1.3)	6 (3.4)	4 (1.1)	11 (3.3)	7 (2.2)	12 (3.4)	44 (2.2)
	19F	6 (4.6)	2 (6.7)	46 (23.5)	27 (18.1)	34 (19.1)	84 (24.1)	27 (8.0)	42 (13.2)	45 (12.8)	313 (15.3)
	23F	13 (9.9)	2 (6.7)	26 (13.3)	15 (10.1)	8 (4.5)	29 (8.3)	20 (5.9)	24 (7.5)	29 (8.2)	166 (8.1)
	**PCV13**										
	3	1 (0.8)	1 (3.3)	2 (1.0)	1 (0.7)	6 (3.4)	12 (3.4)	4 (1.2)	17 (5.3)	9 (2.6)	53 (2.6)
	6A	21 (16.0)	1 (3.3)	13 (6.6)	21 (14.1)	6 (3.4)	2 (0.6)	36 (10.7)	17 (5.3)	16 (4.5)	133 (6.5)
	19A	8 (6.1)	0 (0.0)	14 (7.1)	10 (6.7)	4 (2.2)	7 (2.0)	38 (11.2)	31 (9.7)	26 (7.4)	138 (6.8)
	**PCV15**										
	22F	0 (0.0)	0 (0.0)	0 (0.0)	0 (0.0)	0 (0.0)	3 (0.9)	0 (0.0)	1 (0.3)	0 (0.0)	4 (0.2)
	33F	0 (0.0)	0 (0.0)	0 (0.0)	0 (0.0)	0 (0.0)	0 (0.0)	0 (0.0)	0 (0.0)	0 (0.0)	0 (0.0)
	**PCV20**										
	8	0 (0.0)	0 (0.0)	0 (0.0)	0 (0.0)	0 (0.0)	0 (0.0)	1 (0.3)	0 (0.0)	1 (0.3)	2 (0.1)
	10A	0 (0.0)	0 (0.0)	0 (0.0)	2 (1.3)	0 (0.0)	4 (1.1)	0 (0.0)	0 (0.0)	1 (0.3)	7 (0.3)
	11A	3 (2.3)	1 (3.3)	0 (0.0)	3 (2.0)	0 (0.0)	3 (0.9)	3 (0.9)	2 (0.6)	0 (0.0)	15 (0.7)
	12F	0 (0.0)	0 (0.0)	1 (0.5)	0 (0.0)	0 (0.0)	0 (0.0)	0 (0.0)	0 (0.0)	0 (0.0)	1 (0.05)
	15B	0 (0.0)	0 (0.0)	0 (0.0)	5 (3.6)	0 (0.0)	2 (0.6)	1 (0.3)	0 (0.0)	0 (0.0)	8 (0.4)
	**VRT**	51 (38.9)	7 (23.3)	46 (23.5)	7 (4.7)	1 (0.6)	75 (21.6)	17 (5.0)	13 (4.1)	48 (13.6)	265 (13.0)
	**NVT**	1 (0.8)	3 (10.0)	9 (4.6)	16 (10.7)	8 (4.5)	30 (8.6)	1 (0.3)	12 (3.8)	29 (8.2)	109 (5.3)
	**NT**	2 (1.5)	6 (20.0)	33 (16.8)	2 (1.3)	21 (11.8)	21 (6.0)	16 (4.7)	11 (3.5)	16 (4.5)	128 (6.3)
	**Unknown**	14 (10.7)	0 (0.0)	0 (0.0)	19 (12.8)	57 (32.0)	24 (6.9)	17 (5.0)	51 (16.0)	8 (2.3)	190 (9.3)
	**Total**	131 (100)	30 (100)	196 (100)	149 (100)	178 (100)	348 (100)	338 (100)	318 (100)	352 (100)	2040 (100)

*VRT* Vaccine Related, Types *NVT* Non-Vaccine Types, *NY* Non-Typeable, *PCV* Pneumococcal Conjugate Vaccine. *IPD* Invasive Pneumococcal Disease. *Non-IPD* Non-Invasive Pneumococcal Disease

One thousand and eight (49.4% [95%CI: 47.2–51.6]) isolates were identified from invasive disease studies, 675 isolates (33.1%, [95%CI: 31.1–35.2]) from non-invasive disease studies and 357 isolates from carriage studies (17.5%, [95%CI: 15.9–19.2]). Across all studies, the most common serotypes were 19F (*n* = 313, 15.3% [95%CI: 13.8–17.0]), 23F (*n* = 166, 8.1% [95%CI: 7.0–9.4]), 14 (*n* = 166, 8.1% [95%CI: 7.0–9.4]), 6B (*n* = 163, 8.0% [95%CI: 6.9–9.2]) and 19A (*n* = 138, 6.8% [95%CI: 5.8–7.9]). Non-vaccine types accounted for *n* = 109 (5.3%, [95%CI: 4.4–6.4]) isolates. PCV10, PCV13, PCV15 and PCV20 coverage accounted for 48.4% [95%CI: 46.2–50.6], 64.3% [95%CI: 62.2–66.3], 64.5% [95%CI: 62.4–66.5] and 66.1% [95%CI: 64.0–68.1] respectively. Unknown serotypes made up *n* = 190 (9.3% [95%CI: 8.1–10.7]) of total isolates. Non-typeables made up *n* = 128 (6.3% [95%CI: 5.3–7.4]) of total isolates.

The most common serotypes found in carriage studies were 19F (*n* = 54, 15.1% [95%CI: 11.8–19.2]), 23F (*n* = 41, 11.5% [95%CI: 8.6–15.2]), 6A (*n* = 35, 9.8 [95%CI: 7.1–13.3]) and 19A (*n* = 22, 6.2% [95%CI: 4.1–9.2]) and 6B (*n* = 17, 4.8% [95%CI: 3.0–7.5]). Non-vaccine types accounted for *n* = 13 (3.6%, [95%CI: 2.1–6.1]) isolates. PCV10, PCV13, PCV15 and PCV20 coverage accounted for 33.3% [95%CI: 28.6–38.4], 50.4% [95%CI: 45.3–55.6], 50.4% [95%CI: 45.3–55.6] and 51.8% [95%CI: 46.6–57.0] respectively.

The most common serotypes across non-IPD studies were 19F (*n* = 145, 21.5% [95%CI: 18.5–24.7]), 23F (*n* = 52, 7.7 [95%CI: 5.9–10.0]), 6B (*n* = 38, 5.6% [95%CI: 4.1–7.6]), 6A (*n* = 29, 4.3% [95%CI: 3.0–6.1]) and 1 (*n* = 25. 3.7% [95%CI: 2.5–5.4]). Non-vaccine types accounted for *n* = 44 (6.5% [95%CI: 4.9–8.6]) isolates. PCV10, PCV13, PCV15 and PCV20 coverage accounted for 44.9% [95%CI: 41.2–48.7], 55.1% [95%CI: 51.3–58.8], 55.6% [95%CI: 51.8–59.3] and 58.4% [95%CI: 54.6–62.0] respectively.

The most common serotypes found in IPD studies were 14 (*n* = 142, 14.1% [95%CI: 12.1–16.4]), 19F (*n* = 114, 11.3% [9.5–13.4]), 6B (*n* = 108, 10.7% [95%CI: 9.0–12.8]), 19A (*n* = 95, 14.1% [95%CI: 11.7–16.9]) and 6A (*n* = 69, 6.8% [95%CI 5.4–8.6]). Non-vaccine types accounted for *n* = 42 (4.2% [95%CI: 3.1–5.6]) isolates. PCV10, PCV13, PCV15 and PCV20 coverage accounted for 56.1% [95%CI: 53.0–59.1], 75.3% [95%CI: 72.5–77.9], 75.4% [95%CI: 72.6–78.0] and 76.3% [95%CI: 73.6–78.8] respectively.

Non-typeable serotypes made up *n* = 128 (6.3% [95%CI: 5.3–7.4]) of the total isolates, with carriage studies reporting *n* = 41 (11.5%, [95%CI: 8.6–15.2]) isolates, non-IPD studies reporting *n* = 44 (6.4%, [95%CI: 4.8–8.5]) isolates and IPD studies reporting *n* = 43 (4.3%, [95%CI: 3.2–5.7]) isolates.

**Fig. 2:**
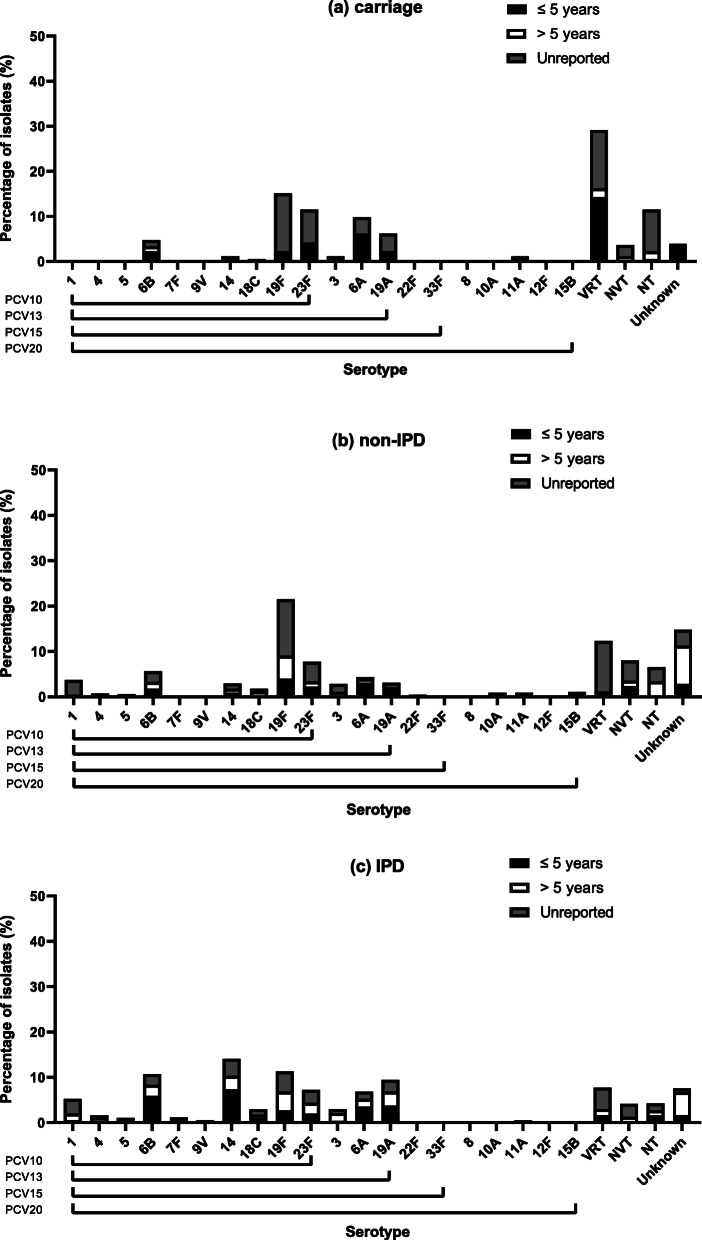
Serotype distributions of *S. pneumoniae* over (**a**) carriage, (**b**) non-invasive disease (non-IPD) and (**c**) invasive disease (IPD) studies. Data shows number of isolates for ≤5 years, > 5 years and unreported (not disclosed or does not fit the age categories). Vaccine-related serotypes represent those that are related to the serotypes found in PCVs. VRT = vaccine-related types, NVT = non-vaccine type, NT = non-typeable. Unknown serotypes are those that were not explicitly reported or those that failed the serotyping process

Figure 2 shows the distributions of the VT serotypes, alongside VRT serotypes and serogroups, as well as the non-typeable strains and non-vaccine types across IPD, non-IPD and carriage studies.

## Discussion

This study aimed to review the epidemiology of pneumococcal serotypes across Malaysia and to calculate the serotype coverage for currently available PCVs and for PCV15 and PCV20 in clinical trials. Currently, there is limited data on serotype prevalence for IPD and non-IPD in the country, and carriage studies are rarely undertaken. Moreover, pneumococcal carriage data from the Malaysian health services are rarely published and therefore, ad hoc studies have taken place in academic centres without a national approach to surveillance. There were limited studies that investigated non-IPD and carriage in children under five years of age, a critical population when studying the epidemiology of the disease. The most common serotypes are all covered by PCV13, with PCV10 covering four of the five most common serotypes across all isolate sources. PCV13 coverage of serotypes from all isolate sources was calculated at 64.3%, lower than a previous estimate [16].

Serotype 14 was shown to be the most common serotype in invasive disease studies when in comparison, carriage studies report very few isolates. Serotype 14 is known to be associated with childhood disease [38]. The distribution of serotypes across the carriage, non-invasive and invasive disease studies may be associated with the age of the subjects and that the reporting of serogroups may not reflect the differences in serotype invasiveness potential, also rank order of serotypes may not also be correlated with invasiveness [39].

Synflorix® contains protein D from non-typeable *Haemophilus influenzae* (NTHi) that acts as a carrier for pneumococcal serotypes, which may offer broader protection against acute otitis media (AOM) [40] as NTHi infection is one of the most common causes of, alongside pneumococcal infection, of AOM in children [41]. PCV13 also offers protection against pneumococcus-related AOM through the immunogenic effects of pneumococcal vaccination [42]. However, an indirect effect of PCV13 implementation is a niche disruption that leads to the increased carriage of NTHi in children [43], but evidence for this is mixed [44]. Synflorix® may also offer cross-reactivity with serotype 19A [45] as shown in a mouse model through a cross-protection mechanism with serotype 19F-specific IgG antibodies [46, 47], although the response to 19A was significantly lower than the 19F response. As we have shown 19A to be a prevalent serotype in both IPD and across all studies, the potential for additional serotype protection is of importance within Malaysia.

Serotype 19A has been seen to increase post-vaccination as described previously [48–51] and has been found to be one of the most prevalent serotypes post-PCV7 implementation globally [52]. Serotype 19A also has a high invasive potential, which can result in severe disease in those who have not been vaccinated against the serotype [53]. The increase in invasive disease caused by non-vaccine types, including 19A not covered by PCV10, might also be related to factors including vaccine coverage across the country, comorbidities, antibiotic usage and carriage of pneumococci [54]. As PCV10 becomes routinely taken within Malaysia, surveillance must be continued to monitor any changes in serotype prevalence which has been seen in other countries, as previously mentioned. Serotype 19A was found to be one of the most prevalent (*n* = 95, 14.1% [95%CI: 11.7–16.9]) in invasive disease studies. This is concerning as the newly implemented PCV10 does not cover 19A, therefore it is possible that a rise in this serotype is seen as other vaccine types reduce. Monitoring of serotype distribution as vaccination becomes widespread is needed to track serotype distribution changes.

Eight of the studies included in this analysis used Quellung reaction as their serotyping technique. Quellung reaction is the gold standard for serotyping; however, due to the high cost of the antisera this method may not be feasible in low-resource settings [55]. Another method used in four of the studies was multiplex PCR, which has the benefit of being cost-effective and easy to implement; however, it can be difficult to differentiate between some specific serotypes among other variants from the serogroup [56].

Non-typeable (NT) pneumococcal isolates formed 6.3% of the total serotypes reported. Typically, NT pneumococci are implicated in non-IPD [57]; this effect was seen in this analysis, with NT isolates making up 6.5% of the total non-IPD serotypes compared to 4.3% of IPD serotypes which suggests non-encapsulated pneumococci are not common for invasive disease cases.

PCV implementation realises several benefits. Studies on the estimated economic impact from PCV introduction in Malaysia [58–60] showed that the introduction of PCV13 into the NIP would be cost-effective and is expected to lead to the reduction in pneumococcal disease burden, as seen with that reported in several countries [31, 61]. Vaccination may also lead to a reduction in antibiotic resistance. Malaysian public and private healthcare providers extensively use antibiotics, especially in the diagnosis of upper respiratory tract infections (49.2%) [62], thus increasing the risk of resistance selection against *S. pneumoniae* as well as other carriage microbiome. Malaysia has shown pneumococcal isolates displaying antibiotic resistance [18, 33], including persistently high penicillin resistance [28] however, PCV vaccination has been shown to reduce resistant serotype prevalence [63].

Many of the studies reviewed were limited in sample size and duration; seven of them had included only isolates from Kuala Lumpur, a highly urbanised area that do not give an accurate representation of the population across the country, thereby failing to offer an accurate representation of pneumococcal epidemiology. There is a need for robust, multi-centre studies, with a large sample size across the country to gain a more accurate representation of the pneumococcal epidemiology. This is especially the case for East Malaysia located on the Borneo island with a different socio-demographic population distribution than Kuala Lumpur in Peninsular Malaysia. Some studies reported serogroups without undergoing further serotyping, and some reported ‘unknown’ serotypes, hampering the reporting of specific serotypes and does not give the full picture of serotype distribution. Unknown serotypes add no value to the study as they cannot be characterised, therefore the vaccine coverage calculations represent the lowest theoretical coverage, as the unknown serotypes were classified as not belonging to the serotypes covered by the PCVs.

Invasive disease sources came from sterile sites, mainly blood and cerebrospinal fluid. Non-invasive disease sources included non-sterile sites, such as nasopharyngeal swabs and sputum. Two studies reported non-invasive isolates from the eye and vagina [26, 30] and three studies included swabs from the ear [26, 28, 30].

The lack of data and the limited number of studies prevented an age analysis from being carried out, nor a study on anatomical site serotypes. Unfortunately, the lack of unbiased studies makes conclusions on epidemiological burden difficult, as the studies included present populations that would not be representative of the country. Selection bias within the studies can give a false picture of serotype distribution as studies may focus on only serotypes from severe cases of disease. Scale, geographical location and the study time differences can affect the representativeness of the data [27]. Studies with more recent collection periods will provide a better insight into current epidemiological trends. Incidence of invasive disease was lacking in the studies. Incidence rates were not able to be calculated with population-based studies as relative risk cannot be calculated when starting with a diseased population and a non-diseased population. A study from 1999 [23] reports the incidence of pneumococcal meningitis at 1.5 cases per 100,000 in the Malaysian population, but no other articles included in this study report invasive pneumococcal disease incidence.

Many studies in our analysis included isolates from hospitals or medical centres. Although hospitals provide cohorts of individuals who might have IPD or non-IPD, the population does not truly reflect the country demographics. Potential limitations with hospital-based studies include difficulties in finding control groups, risk of results duplication if patients move between hospitals and difficulties in defining the population. This study has highlighted the need for carriage studies within Malaysia. In the past 20 years, only one small-scale study on daycare children in Kuala Lumpur has been reported [32]. Most other studies were clinically related, with only two multi-centre surveillance studies reported by the Institute for Medical Research (IMR) involving patient cohorts from 2008 to 2009 and 2014–2017, respectively [13, 30]. Unfortunately, no further data is available on childhood carriage from other geographical regions in Malaysia. In fact, the paucity of such studies and the need for pneumococcal carriage surveillance in Malaysia has been clearly highlighted [64]. Pneumococcal surveillance programmes are not only needed to assess the pneumococcal burden and the progress of immunisation initiative but also alerts the health bodies and scientific community on changes in serotype distribution and offers a view into herd immunity effects in the population [64–67]. The implementation of a pneumococcal surveillance programme is vital for the understanding of pneumococcal population dynamics in Malaysia, especially in children under five years of age as we are now into the post-PCV era following implementation of PCV10 into the routine NIP [16].

## Conclusion

This study provides a comprehensive insight into the all-inclusive pneumococcal serotype prevalence in Malaysia since 1989 before the introduction of PCV10 under the Malaysian NIP in late 2020. Our results highlight the need for continued surveillance of pneumococcal seroepidemiology and disease prevalence, as it is difficult to accurately estimate the disease burden and serotype coverage of pneumococcal vaccines. New conjugate vaccines in development such as PCV15 and PCV20 will offer broader serotype coverage, but whether these would confer a significant added protective efficacy over the existing pneumococcal vaccine to the Malaysian population would need continued monitoring, especially in the coming years following a potential serotype shift in the post-PCV era as observed in many other countries.

## Data Availability

All data generated or analysed during this study are included in this published article.

## Abbreviations

IPD: Invasive pneumococcal disease
LMIC: Low and middle-income countries
Non-IPD: Non-invasive pneumococcal disease
NT: Non-typeable
NTHi: Non-typeable *Haemophilus influenzae*
NVT: Non-vaccine type
PCV: Pneumococcal conjugate vaccine
VT: Vaccine type
